# Predicted 10-year cardiometabolic risk among hospital staff: a cross-sectional study

**DOI:** 10.3389/fpubh.2026.1929290

**Published:** 2026-09-16

**Authors:** Ching-Yuan Wu, Yen-Wen Wu, Hon-Yen Wu, Jia-Ren Tsai, Chiung-Fang Chang, Yen-Ling Chiu

**Affiliations:** 1 Department of Medical Research, Far Eastern Memorial Hospital, New Taipei City, Taiwan; 2 Graduate Institute of Medicine, Yuan-Ze University, Taoyuan, Taiwan; 3 Division of Cardiology, Cardiovascular Medical Center Far Eastern Memorial Hospital, New Taipei City, Taiwan; 4 Department of Internal Medicine, Far Eastern Memorial Hospital, New Taipei City, Taiwan; 5 School of Medicine, National Yang Ming Chiao Tung University, Taipei, Taiwan; 6 Institute of Epidemiology and Preventive Medicine, College of Public Health, National Taiwan University, Taipei City, Taiwan; 7 Department of Internal Medicine, National Taiwan University Hospital and College of Medicine, Taipei, Taiwan; 8 Department of Statistics and Information Science, Fu Jen Catholic University, New Taipei City, Taiwan; 9 Department of Health Management, Far Eastern Memorial Hospital, New Taipei City, Taiwan; 10 Graduate Institute of Clinical Medicine, College of Medicine, National Taiwan University, Taipei City, Taiwan

**Keywords:** cardiometabolic health, chronic disease risk assessment, decision tree, nursing workforce, occupational health

## Abstract

**Objective:**

Chronic diseases and cardiometabolic disorders are major public health and occupational health challenges in Taiwan, particularly among healthcare workers exposed to shift work, high occupational stress, and irregular schedules. Nurses and hospital staff are especially vulnerable to hypertension, type 2 diabetes mellitus, stroke, and cardiovascular disease risk. Using a localized digital risk assessment platform and machine learning–based data-driven modeling, this study aimed to evaluate chronic disease risk among medical center employees and identify high-risk occupational subgroups for precision health management.

**Participants/Methods:**

This retrospective cross-sectional study included 1,916 employees aged 35–70 at a Northern Taiwan medical center from 2019 to 2024. Ten-year predicted risks for coronary artery disease, stroke, type 2 diabetes mellitus, hypertension, and major adverse cardiovascular events were estimated using the Chronic Disease Risk Assessment Platform. To assess associations between predicted risk and demographic or occupational variables, we used ordinal logistic regression, generalized linear models, and decision tree algorithms.

**Results:**

Hypertension exhibited the highest overall predicted risk (38.6%), followed by type 2 diabetes mellitus and MACE. Male employees had higher odds of being classified into a higher predicted HTN risk category than female employees (odds ratio [OR] = 4.18, 95% confidence interval [CI]: 3.21–5.44, *p* < 0.001). By occupational category, nurses had higher odds of being in a higher predicted 10-year HTN risk category than administrative staff (OR = 1.32, 95% CI: 1.04–1.68, *p* = 0.024). Multivariable generalized linear models identified increasing age and metabolic factors, including obesity, elevated blood pressure, hyperglycemia, and dyslipidemia, as the major predictors of predicted cardiometabolic risk, whereas occupational category was not independently associated with disease risk after adjustment. Significant interaction effects were observed for CAD, stroke, HTN, and MACE. Ten-fold cross-validated decision tree models identified age, waist circumference, blood pressure, and body mass index as key classification variables, with overall accuracies ranging from 86.9 to 91.7% and F1-scores ranging from 0.622 to 0.936 across the five outcomes.

**Conclusion:**

This study applied disease risk prediction models developed by the Taiwan Health Promotion Administration platform, integrating clinical indicators and machine learning analyses to identify healthcare workers at increased disease risk. The findings suggest that localized digital prediction tools may be integrated into institutional health surveillance systems to support continuous health monitoring, early risk identification, and proactive preventive care in healthcare settings.

## 1 Introduction

Cardiovascular disease (CVD) remains the leading cause of morbidity and mortality worldwide, contributing substantially to global deaths and healthcare burden (1, 2). Major adverse cardiovascular events (MACE), including myocardial infarction, stroke, and cardiovascular death, represent the most severe clinical consequences of CVD. Since the concept of cardiovascular risk factors was established by the landmark Framingham Heart Study, numerous epidemiological studies have identified key determinants such as hypertension (HTN), type 2 diabetes mellitus (T2DM), dyslipidemia, smoking and obesity, which collectively accelerate the development of atherosclerosis and increase the risk of cardiovascular complications (3). Among these, HTN is strongly associated with cerebrovascular events, while T2DM contributes to endothelial dysfunction, vascular injury, and increased cardiovascular risk (4). Recently, the American Heart Association introduced the concept of cardiovascular-kidney-metabolic (CKM) syndrome, highlighting the interconnected relationships among metabolic disorders, chronic kidney disease, and cardiovascular disease across the disease continuum (5, 6). This framework highlights the importance of integrated risk assessment and early prevention strategies targeting clustered cardiometabolic risk factors. Therefore, understanding the combined and interactive effects of cardiometabolic conditions is essential for improving cardiovascular risk stratification and preventive healthcare strategies.

Chronic noncommunicable diseases are a major global public health concern, representing approximately 74% of deaths globally (7, 8). Occupational and lifestyle-related factors may further contribute to the accumulation of cardiometabolic risk among working populations. Previous studies have demonstrated that demographic characteristics, metabolic abnormalities, and unhealthy lifestyle behaviors, such as smoking, alcohol consumption, and obesity, are strongly associated with cardiometabolic disorders, including HTN, DM, and CVD (9). In particular, the global burden of DM is rapidly increasing, affecting approximately 529 million individuals in 2021 and projections reaching over 1.31 billion by 2050 (10). Furthermore, DM and pre-DM are highly prevalent among individuals with HTN, highlighting the close interrelationship between these metabolic disorders and their synergistic effect on cardiovascular risk (11). Cardiometabolic conditions, including HTN, DM, and CVD, represent a significant and persistent public health concern, driven by unhealthy lifestyle behaviors, population aging, and rising socioeconomic pressures on healthcare systems (12). Taiwan is no exception, with chronic diseases ranking among its leading causes of death (13). Nevertheless, large-scale studies utilizing health examination databases to evaluate cardiometabolic risk factors remain limited in this country, particularly in Asian populations.

Healthcare workers are a unique occupational group with increased susceptibility to cardiometabolic diseases (14). Elevated occupational stress and burnout, which reportedly affect up to 70% of healthcare workers, reflect a substantial and multifactorial burden arising from work-related demands, personal life pressures, and their interactions. A combination of these stressors may adversely affect overall health, potentially leading to increased cardiometabolic risk (15). Nurses, in particular, frequently experience rotating and night shift work, disrupting their circadian rhythms and increasing the risk of HTN, metabolic disorders, and T2DM (16, 17). Furthermore, current clinical guidelines recommend early screening for DM or pre-DM in high-risk populations, including individuals with overweight or obesity and additional risk factors (18). Therefore, early identification and management of cardiometabolic risks are essential among healthcare workers.

Multiple modifiable risk factors, including obesity, smoking, HTN, T2DM, dyslipidemia, and CKD, contribute to cardiovascular and other chronic disease risks and may have cumulative or interacting effects (19–22). Sex-related differences in cardiovascular outcomes have also been reported, with females experiencing a higher risk of adverse events following acute coronary syndrome (23). Together, these findings highlight complex and interacting behavioral and clinical risk factors, rather than isolated conditions, as the antecedent of cardiometabolic diseases (24, 25). In the present study, the predicted 10-year risks of five cadiometabolic outcomes- CAD, stroke, HTN, T2DM, and MACE-were investigated to comprehensively capture both chronic conditions and adverse clinical outcomes across the cardiometabolic spectrum. Therefore, an integrated analytical approach that considers the coexistence and interaction of multiple risk factors across different subgroups is required to identify high-risk populations.

Studies of Taiwanese hospital employees have reported occupational differences in metabolic syndrome and associations with work-related factors, including burnout, night-shift work, and job seniority (26–28). Moreover, an 8-year cohort study of 5,775 Taiwanese hospital workers found that night-shift work was associated with an increased risk of metabolic syndrome (29). However, previous Taiwanese studies have primarily focused on metabolic syndrome or specific occupational exposures, leaving the broader cardiometabolic risk profiles of hospital employees less well characterized.

Digital health is increasingly being harnessed to improve the continuity of care and management of chronic diseases. Risk assessment platforms that integrate routinely collected demographic, behavioral, and clinical information may facilitate the identification and stratification of individuals with elevated predicted cardiometabolic risk (30–32). However, the application of such platform-derived risk estimates to simultaneously characterize multiple cardiometabolic risk profiles among healthcare workers, particularly across different occupational groups, remains insufficiently studied. Accordingly, the additional statistical and decision tree analyses in this study were not intended to develop or validate a new disease prediction model. Rather, they were applied to characterize associations and risk-stratification patterns within the platform-derived risk estimates and to examine how these patterns varied across demographic, clinical, and occupational subgroups.

To address this gap, this study used routine employee health examination data from a large tertiary medical center in northern Taiwan to characterize the predicted 10-year risks of five cardiometabolic outcomes—CAD, stroke, T2DM, HTN, and MACE—and to examine their associations with demographic, behavioral, clinical, and occupational factors. Using complementary ordinal logistic regression, generalized linear models, and CART analyses, we further examined differences in predicted risk patterns across occupational groups and identified subgroups with higher platform-derived predicted risk.

## 2 Methods

### 2.1 Study design and data source

This retrospective cross-sectional study was conducted at a medical center in Northern Taiwan and was approved by the Institutional Review Board of Far Eastern Memorial Hospital (FEMH IRB No. 113024-F). The requirement for informed consent was waived by the Institutional Review Board because all data used in this study were de-identified prior to analysis, thereby protecting participant privacy and confidentiality. The study was conducted in accordance with the ethical principles of the Declaration of Helsinki and relevant institutional and regulatory guidelines. Data were collected from the hospital employee health examination database, which contains employee health examination recorded between 2019 and 2024.

### 2.2 Study population

Employees aged between 35 and 70 years who had completed at least one comprehensive health examination during the study period were included. A total of 3,430 health examination records were initially retrieved. Records with incomplete data required for the study analyses were excluded (*n* = 88). To avoid repeated measurements, we retained only each employee’s most recent examination record. After exclusion of incomplete records and retention of the most recent examination for employees with repeated health examinations, the final study cohort comprised 1,916 individuals. All data were de-identified before analysis to ensure participant confidentiality and data security. Individuals diagnosed with a specific chronic disease before or during the data collection period were excluded from the corresponding disease-specific analysis because the study aimed to estimate the 10-year risk of developing that disease among individuals without the disease. Consequently, the analytic sample size differed across the five disease-specific analyses. These disease-specific exclusions were based on pre-existing disease status rather than missing data. Figure 1 presents the participant selection process and the final sample sizes for each disease analysis.

**Figure 1:**
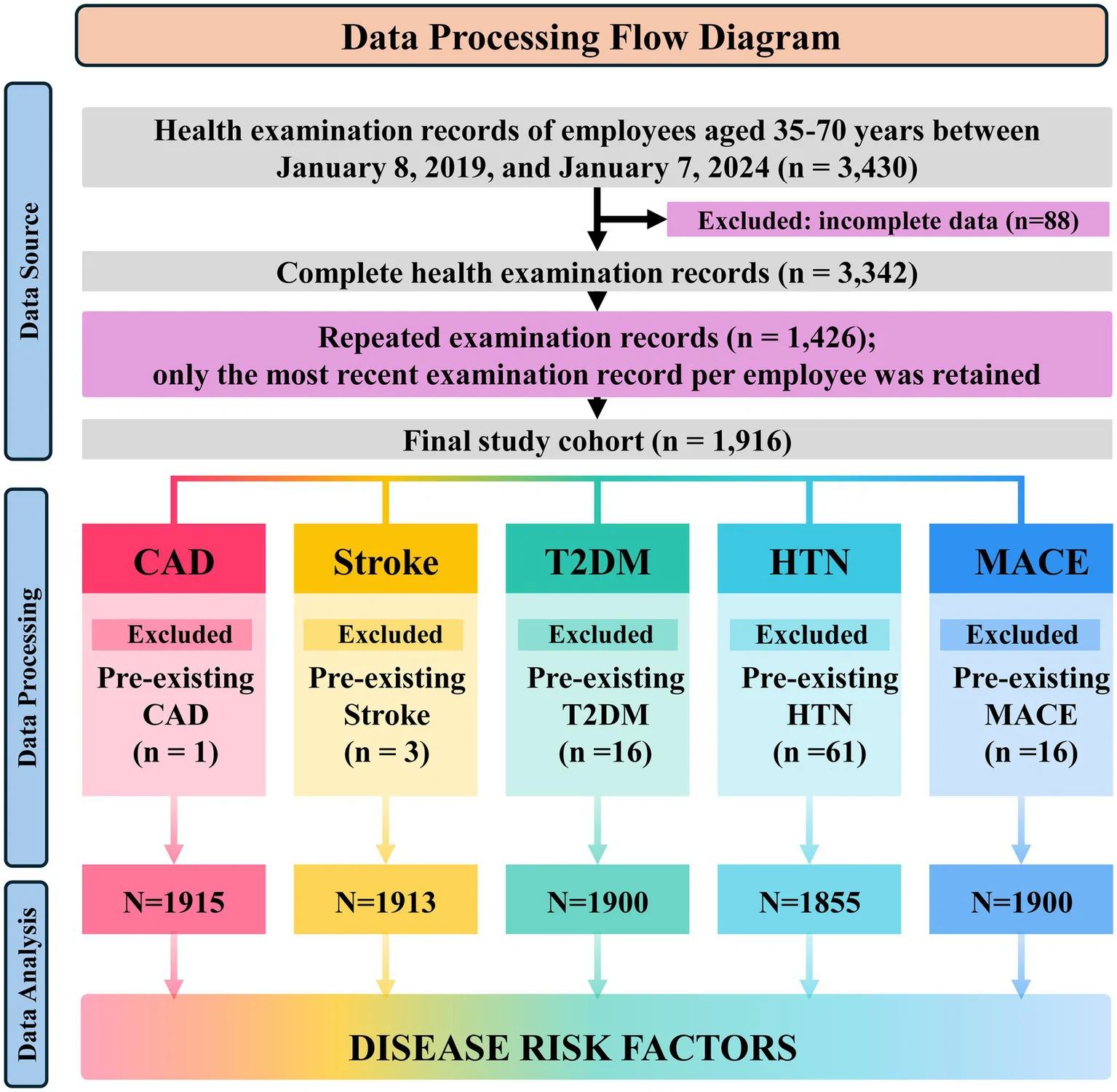
Flow diagram of participant inclusion and data processing. CAD, coronary artery disease; T2DM, Type 2 diabetes mellitus; HTN, hypertension; MACE, major adverse cardiovascular events. Participants with pre-existing disease were excluded from the corresponding disease-specific risk analyses to avoid reverse causation.

### 2.3 Dependent and predictor variables

The estimated 10-year risks of the five major chronic diseases (CAD, stroke, T2DM, HTN, and MACE) were the study’s primary dependent variables. These risk estimates were obtained from the Chronic Disease Risk Assessment Platform, which was jointly developed by the Health Promotion Administration and the National Health Research Institutes in Taiwan (33). Relevant participant data from the employee health examination database were entered into the platform to generate individual disease-specific 10-year risk estimates for CAD, stroke, T2DM, HTN, and MACE. The predicted risk scores were generated as continuous percentage values (%), representing the estimated 10-year probability of developing each disease. For statistical analyses, the risk scores were categorized into three ordinal risk levels according to the platform criteria: low risk (<10%), intermediate risk (10 ~ 20%), and high risk (≥20%). The categorized risk levels were used as outcomes in the ordinal logistic regression analyses, whereas the continuous risk scores (%) were used in the generalized linear model analyses.

Derived from the hospital employee health examination database, predictor variables included demographic characteristics, physical examinations, anthropometric measurements, biochemical parameters, health behaviors, comorbidity history, and occupational categories. Age and sex were the demographic variables. Anthropometric indicators comprised BMI and waist circumference. Biochemical parameters consisted of blood pressure, fasting glucose, total cholesterol (TC), high-density lipoprotein cholesterol (HDL-C), low-density lipoprotein cholesterol (LDL-C), triglycerides (TG), and the TC/HDL-C ratio as part of routine health examination. Anthropometric measurements and blood pressure were obtained during routine employee health examinations using standardized procedures. Blood samples were analyzed by the medical center’s clinical laboratory using validated methods with established internal quality-control procedures. For categorical analyses, elevated systolic blood pressure was defined as SBP ≥ 140 mmHg, elevated fasting glucose as ≥126 mg/dL, elevated TC as ≥240 mg/dL, low HDL-C as <40 mg/dL, elevated LDL-C as ≥160 mg/dL, elevated TG as ≥200 mg/dL, and high waist circumference as ≥90 cm in male and ≥80 cm in female. Information on health behaviors included smoking status, which was categorized as current smoker or non-current smoker based on self-reported information obtained during the routine health examination. For comorbidity history, T2DM and HTN were recorded. Occupational categories were also included as an important variable. Information on medication use was not available in the employee health examination database and was therefore not included in the analyses. Occupational categories were classified according to employees’ primary professional roles at the medical center into four groups: physicians, nursing staff, allied health professionals, and administrative staff. Physicians included attending and resident physicians; nursing staff comprised nursing personnel; allied health professionals included pharmacists, respiratory therapists, therapists, medical technologists, dietitians, radiologic technologists, social workers, technicians, and audiologists; and administrative staff included research personnel, administrative personnel, and information technology personnel, according to the hospital’s occupational classification system. All these variables served as candidate predictors in the risk prediction analyses. All these variables were considered candidate predictors in the chronic disease risk analyses.

### 2.4 Statistical analysis

All data were analyzed using IBM SPSS Statistics (version 26.0), with data visualization conducted in GraphPad Prism (version 11). All tests were two-tailed, and a *p*-value below 0.05 indicated statistical significance. For each disease, the mean predicted 10-year risk was calculated as the arithmetic mean of the individual predicted risk scores (%) across all participants. To assess potential selection bias associated with the exclusion of incomplete records, data completeness was compared by sex and occupational category using Pearson’s chi-square tests. When a significant overall difference was identified across occupational categories, *post-hoc* pairwise comparisons were performed with Benjamini–Hochberg false discovery rate (FDR) correction.

Associations between categorized chronic disease risk levels (low, intermediate, and high risk) and candidate predictor variables were examined using ordinal logistic regression (OLR), with results presented as adjusted odds ratios (aORs) and 95% confidence intervals (CIs). To evaluate differences in continuous disease risk scores (%) across occupational groups while adjusting for age and sex, generalized linear models (GLMs) were applied, and estimated marginal means with 95% confidence intervals were calculated. Multicollinearity was evaluated using variance inflation factors (VIFs), with all VIF values below 2.0, indicating negligible multicollinearity among the predictor variables. Interaction terms were additionally evaluated in the multivariable GLMs to examine potential effect modification among demographic, metabolic, and occupational factors. To account for multiple testing in the interaction analyses, *p* values were adjusted using the Benjamini–Hochberg false discovery rate (FDR) procedure, with an FDR-adjusted *p* value < 0.05 considered statistically significant.

Decision tree analysis using the Classification and Regression Trees (CART) algorithm was also conducted to identify key predictors associated with higher chronic disease risk levels. For CART analyses, disease risk categories were dichotomized into low-risk (category 1) and higher-risk groups (categories 2 and 3 combined). Intermediate- and high-risk participants were merged because the number of individuals in the high-risk category was relatively small, which could adversely affect model stability and classification performance. Therefore, the CART model was designed to identify factors associated with membership in the higher-risk group. These terminal-node values represent subgroup proportions of higher-risk classification rather than individual risk probabilities. All available candidate predictors, including demographic characteristics, anthropometric measurements, biochemical parameters, health behaviors, comorbidity history, and occupational categories, were entered into the CART models. Internal validation of the CART models was performed using 10-fold cross-validation. Model performance was evaluated using overall classification accuracy and class-sensitive measures, including sensitivity, specificity, precision, and F1-score, with the combined intermediate/high-risk group defined as the positive class and the low-risk group as the negative class. Predictor importance and tree structures were generated automatically by the algorithm to identify the most influential variables associated with higher disease risk.

Because the dependent variables in the generalized linear models were the predicted 10-year disease risks generated by the National Health Administration Chronic Disease Risk Assessment Platform, which incorporates several clinical variables (including blood pressure, fasting glucose, blood lipids, and BMI) into its prediction algorithm, the estimated regression coefficients for these variables should be interpreted as reflecting the sensitivity of the platform-derived risk scores to their constituent predictors rather than as independent causal associations. Accordingly, the GLM analyses were intended primarily to characterize the relative contribution of candidate variables to the predicted risk estimates rather than to infer causal relationships.

## 3 Results

### 3.1 Demographic and occupational profiles of the study cohort and sex-based differences

A total of 3,430 health examination records were initially retrieved, of which 88 (2.57%) were excluded because of incomplete data. The proportion of incomplete records did not differ significantly by sex (male, 2.70%; female, 2.52%; *p* = 0.764), but differed modestly across occupational categories (*p* = 0.028) (Supplementary Table S1A). *Post-hoc* pairwise comparisons with FDR correction showed a higher proportion of incomplete records among nursing staff than administrative/other staff (3.27% vs. 1.05%; FDR-adjusted *p* = 0.030) (Supplementary Table S1B), whereas no other pairwise comparisons remained statistically significant after correction.

After exclusion of incomplete records and repeated health examination records, the final study cohort included 1,916 employees from a medical center in Northern Taiwan (Table 1; Figure 1). After excluding participants with pre-existing disease for each disease-specific analysis, the final analytical sample sizes were 1,915 for CAD, 1,913 for stroke, 1,900 for T2DM, 1,855 for HTN, and 1,900 for MACE (Figure 1). Female participants outnumbered male participants (75.3% vs. 24.7% males). Furthermore, most of the participants were aged 35 ~ 49 years (73.3%, *n* = 1,404), followed by those aged 50 ~ 59 years (20.5%) and 60–70 years (6.2%). The overall mean age was 45.5 ± 7.7 years. Males had a significantly higher mean age than females (46.8 ± 8.7 vs. 45.1 ± 7.3 years, *p* < 0.001) (Table 1). Regarding occupational categories, nursing staff represented the largest proportion (43.1%), followed by other health professionals (20.1%), physicians (19.2%), and administrative and other personnel (17.6%). Occupational distribution significantly differed between sexes (*p* < 0.001) (Table 1).

**Table 1:** Baseline characteristics of the study population according to sex (*n* = 1,916).

Variables	Total (*n* = 1,916)	Male (*n* = 474)	Female (*n* = 1,442)	*p*-value
Demographic characteristics				
Age, years	45.5 ± 7.7	46.8 ± 8.7	45.1 ± 7.3	<0.001
Age, *n* (%)				<0.001
35–49	1,404 (73.3)	317 (66.9)	1,087 (75.4)	
50–59	393 (20.5)	108 (22.8)	285 (19.8)	
60–70	119 (6.2)	49 (10.3)	70 (4.9)	
Occupational category, *n* (%)				<0.001
Physicians	368 (19.2%)	273 (57.6%)	95 (6.6%)	
Nursing staff	826 (43.1%)	16 (3.4%)	810 (56.2%)	
Allied health professionals	384 (20.0%)	122 (25.7%)	262 (18.2%)	
Administrative and other staff	338 (17.6%)	63 (13.3%)	275 (19.1%)	
BMI, kg/m^2^	24.2 ± 4.2	25.5 ± 3.4	23.8 ± 4.3	<0.001
BMI category, *n* (%)				<0.001
Underweight*	62 (3.2)	4* (0.8)	58 (4.0)	
Normal weight*	999 (52.1)	165 (34.8)	834 (57.8)	
Overweight*	441 (23.0)	170 (35.9)	271 (18.8%)	
Obesity*	414 (21.6)	135 (28.5)	279 (19.4)	
Clinical measurements‡				
SBP (mmHg)	119.5 ± 18.5	125.3 ± 15.9	117.6 ± 18.8	<0.001
DBP (mmHg)	71.0 ± 12.6	75.7 ± 11.4	69.4 ± 12.6	<0.001
Clinical conditions, *n* (%)				
Hypertension**	327 (17.1%)	113 (23.8%)	214 (14.8%)	<0.001
Laboratory measurements‡				
Fasting blood glucose, mg/dL	91.9 ± 19.6	96.2 ± 20.8	90.5 ± 19.0	<0.001
Diabetes mellitus†, *n* (%)	69 (3.6%)	29 (6.1%)	40 (2.8%)	<0.001
Triglycerides, mg/dL	102.1 ± 69.7	130.8 ± 91.0	92.7 ± 58.2	<0.001
HDL-C, mg/dL	58.8 ± 14.4	50.0 ± 11.6	61.7 ± 14.0	<0.001
LDL-C, mg/dL	111.9 ± 30.3	116.4 ± 33.0	110.4 ± 29.2	<0.001

Values are presented as mean ± standard deviation or number (percentage).

*Underweight: BMI < 18.5 kg/m^2^; Normal weight: 18.5 kg/m^2^ ≤ BMI < 24 kg/m^2^; Overweight: 24 kg/m^2^ ≤ BMI < 27 kg/m^2^; Obesity: BMI ≥27 kg/m^2^; The male underweight subgroup included only four participants; therefore, estimates for this subgroup should be interpreted with caution.

**Hypertension defined as SBP ≥140 mmHg, DBP ≥90 mmHg, or a history of hypertension.

†Diabetes mellitus defined as fasting blood glucose ≥126 mg/dL or a history of diabetes mellitus.

‡Cutoffs for categorized clinical and anthropometric variables were defined as follows: elevated SBP, ≥140 mmHg; elevated fasting glucose, ≥126 mg/dL; elevated TC, ≥240 mg/dL; low HDL-C, <40 mg/dL; elevated LDL-C, ≥160 mg/dL; elevated TG, ≥200 mg/dL; and high waist circumference, ≥90 cm in male and ≥80 cm in female.

BMI, body mass index; SBP, systolic blood pressure; DBP, diastolic blood pressure. TC, total cholesterol; HDL-C, high-density lipoprotein cholesterol; LDL-C, low-density lipoprotein cholesterol.

Regarding health status, the overall mean BMI was 24.2 ± 4.2 kg/m^2^. Notably, 44.6% of employees were classified as overweight or obese, indicating a substantial burden of weight-related chronic disease risk that warrants further attention and intervention. The mean systolic blood pressure (SBP) and diastolic blood pressure (DBP) were 119.5 ± 18.5 and 71.0 ± 12.6 mmHg, respectively. Both SBP and DBP were significantly higher in males than in females (*p* < 0.001) (Table 1). Additionally, 17.1% of employees had hypertension, with a significantly higher prevalence among males (23.8%) than females (14.8%) (*p* < 0.001).

For glycemic parameters, the overall mean fasting glucose level was 91.9 ± 19.6 mg/dL. Male employees had a significantly higher mean fasting glucose level (96.2 mg/dL) than females (90.5 mg/dL) (*p* < 0.001). Overall, 3.6% of employees had T2DM. Regarding lipid profiles, the mean triglyceride, HDL-C, and LDL-C levels (102.1 ± 69.7, 58.8 ± 14.4, and 111.9 ± 30.3 mg/dL, respectively) also demonstrated significant sex differences. Male employees had significantly higher TG levels than females (*p* < 0.001). Conversely, HDL-C levels were significantly higher in females (*p* < 0.001), while LDL-C levels were significantly higher in males (*p* < 0.001).

Overall, the study population predominantly included middle-aged female employees; however, cardiometabolic profile was consistently less favorable among male employees. Specifically, males demonstrated significantly higher age, BMI, blood pressure, fasting glucose, triglyceride, and LDL-C levels, along with lower HDL-C levels, than females. In addition, HTN was notably more prevalent among males. Therefore, a substantial proportion of healthcare workers, especially males, appear to be at increased risk for cardiometabolic disorders.

### 3.2 Predicted 10-year risk of five major chronic diseases among employees

The mean predicted 10-year risk among hospital employees was highest for HTN (38.55%), followed by T2DM (21.88%), MACE (10.34%), stroke (7.33%), and CAD (6.29%) (Figure 2A). In the unadjusted comparison of continuous predicted risk scores between sexes, no significant difference in HTN risk was observed. However, male employees exhibited significantly higher risks than females for the other four diseases (all, *p* < 0.001).

**Figure 2:**
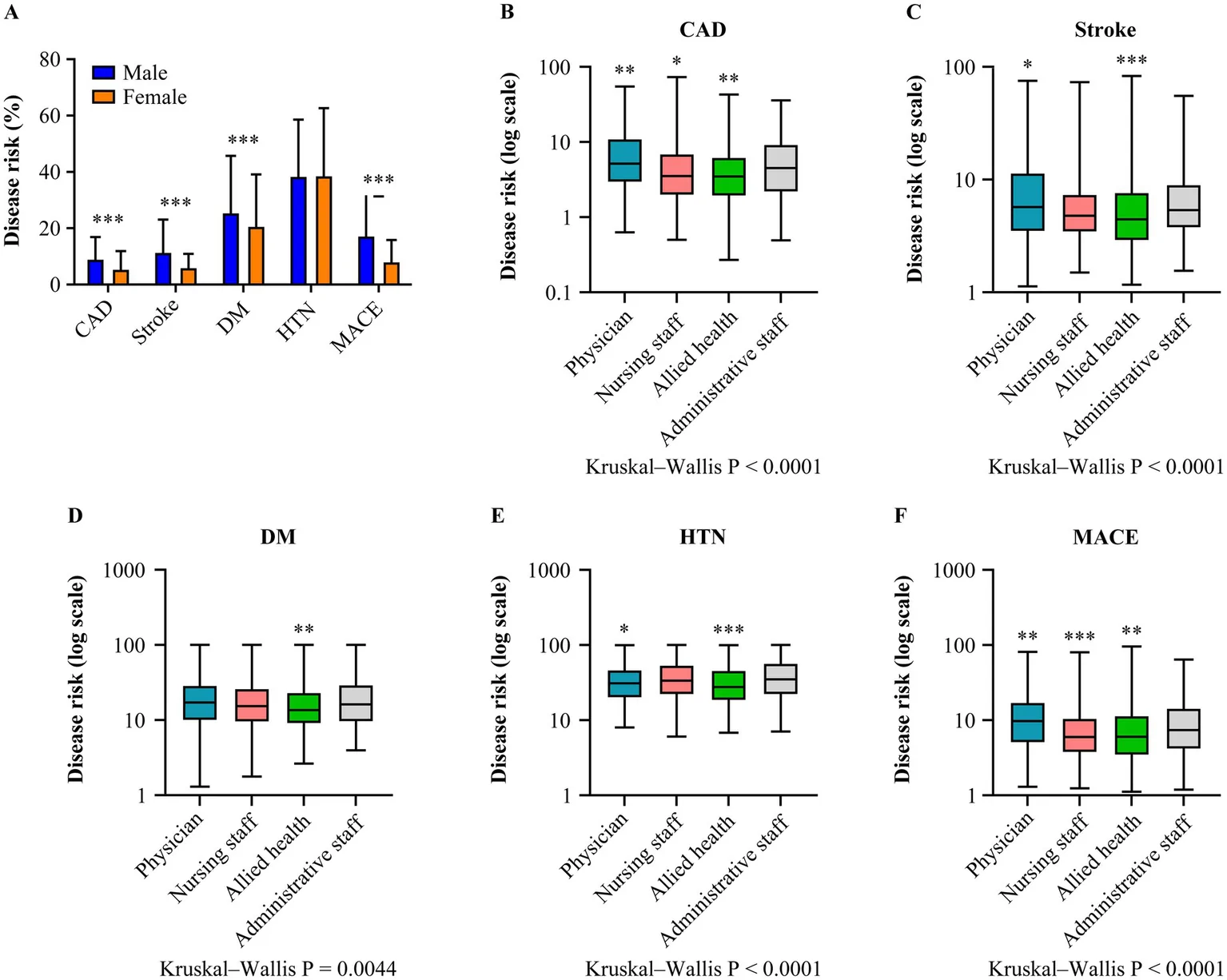
Predicted cardiometabolic risk by sex and occupation. **(A)** Mean predicted risk of CAD, stroke, T2DM, HTN, and MACE by sex. **(B–F)** Unadjusted distribution of predicted risk across occupational groups for each disease. Data are presented as boxplots on a logarithmic scale. Group differences were assessed using the Kruskal–Wallis test. *p < 0.05, **p < 0.01, ***p < 0.001. CAD, coronary artery disease; T2DM, type 2 diabetes mellitus; HTN, hypertension; MACE, major adverse cardiovascular events.

Figures 2B–F presents the unadjusted distribution of predicted disease risk across occupational groups, including physicians, nursing staff, allied health professionals, and administrative staff. These analyses represent unadjusted descriptive comparisons without accounting for potential covariates. Overall, the distribution of predicted disease risk significantly differed among occupational categories for all five major disease outcomes. For CAD (Figure 2B), risk distributions differed significantly across occupations (Kruskal–Wallis test, *p* < 0.0001). Physicians, nursing staff, and allied health professionals showed significantly different predicted risk levels compared with that of the administrative staff. Similarly, stroke risk (Figure 2C) varied significantly among occupational groups (*p* < 0.0001); predicted risk levels were significantly higher among physicians and significantly lower among allied health professionals than among administrative staff.

In T2DM (Figure 2D), a modest but statistically significant difference was observed (*p* = 0.0044). Allied health professionals exhibited a significantly lower unadjusted predicted risk than the administrative staff. For HTN (Figure 2E), significant differences were identified across occupations (*p* < 0.0001). Compared with the administrative staff, physicians and allied health professionals showed significantly lower unadjusted predict risks. Likewise, the unadjusted predicted risk of MACE (Figure 2F) differed significantly among occupational groups (*p* < 0.0001). In particular, nursing staff and allied health professionals demonstrated significantly higher unadjusted predicted risks than the administrative staff. Overall, the unadjusted descriptive comparisons showed significant differences in predicted risk distributions across occupational groups for all five cardiometabolic outcomes.

### 3.3 Multivariable and interaction analyses of predicted cardiometabolic disease risks

In the multivariable generalized linear models, increasing age was consistently associated with significantly higher predicted risks across all five cardiometabolic outcomes (Table 2). Compared with participants aged 35–49 years, those aged 50–59 and 60–70 years exhibited progressively higher predicted risks for CAD, stroke, T2DM, HTN, and MACE. Male sex was associated with higher predicted risks of CAD, stroke, and MACE, whereas lower predicted risks were observed for T2DM and HTN after adjustment for covariates. Occupational category was not independently associated with any of the five outcomes after multivariable adjustment. Compared with normal-weight participants, obesity was consistently associated with markedly higher predicted risks across all outcomes, whereas underweight status was associated with lower predicted risks. In addition, high waist circumference, elevated systolic blood pressure, elevated fasting glucose, elevated triglycerides, elevated LDL cholesterol, and current smoking were independently associated with increased predicted risks for most cardiometabolic outcomes, although the magnitude of these associations varied across diseases. Among all predictors, elevated fasting glucose demonstrated the strongest association with T2DM risk (*β* = 52.24, 95% CI: 49.62–54.86), while elevated systolic blood pressure showed the strongest association with HTN risk (*β* = 30.34, 95% CI: 28.59–32.09). These large effect sizes should be interpreted as reflecting the sensitivity of the predicted risk scores to their input indicators, rather than independent causal associations. To evaluate the robustness of the multivariable findings, univariable generalized linear models were also performed (Supplementary Table S2). The results were generally consistent with those of the multivariable analyses. The univariable analyses showed patterns similar to those observed in the multivariable models, although the magnitude of several associations was attenuated covariate adjustment.

**Table 2:** Multivariable generalized linear models for predicted cardiometabolic disease risks.

Variable	CAD *β* (95% CI) *p* value	Stroke *β* (95% CI) *p* value	T2DM *β* (95% CI) *p* value	HTN *β* (95% CI) *p* value	MACE *β* (95% CI) *p* value
Demographic factors					
Age					
Age 50–59	5.67 (5.22, 6.13) <0.001	5.03 (4.52, 5.54) <0.001	7.03 (5.96, 8.09) <0.001	16.99 (15.61, 18.38) <0.001	7.32 (6.68, 7.95) <0.001
Age 60–70	17.17 (16.38, 17.94) <0.001	15.74 (14.86, 16.61) <0.001	13.60 (11.73, 15.47) <0.001	29.55 (27.08, 32.02) <0.001	20.57 (19.46, 21.67) <0.001
Sex					
Male	0.97 (0.39, 1.55) 0.001	3.19 (2.55, 3.84) <0.001	−2.65 (−4.00, −1.30) <0.001	−7.86 (−9.60, −6.13) <0.001	6.13 (5.32, 6.94) <0.001
Occupation					
Physician	0.70 (0.02, 1.37) 0.044	0.24 (−0.52, 0.99) 0.537	0.34 (−1.25, 1.93) 0.676	0.24 (−1.80, 2.29) 0.816	−0.42 (−1.36, 0.53) 0.389
Nurse	−0.05 (−0.57, 0.47) 0.845	0.29 (−0.29, 0.86) 0.329	−0.37 (−1.58, 0.85) 0.554	0.49 (−1.06, 2.05) 0.534	−0.32 (−1.05, 0.40) 0.380
Allied health professionals	−0.53 (−1.12, 0.07) 0.081	−0.08 (−0.74, 0.59) 0.826	−0.48 (−1.87, 0.92) 0.503	−1.23 (−3.02, 0.56) 0.178	−0.76 (−1.59, 0.07) 0.073
Anthropometric measure					
Underweight	−1.48 (−2.51, −0.45) 0.005	−1.21 (−2.36, −0.05) 0.040	−4.71 (−7.12, −2.30) <0.001	−7.93 (−11.00, −4.86) <0.001	−1.66 (−3.10, −0.22) 0.024
Overweight	1.36 (0.87, 1.86) <0.001	0.51 (−0.04, 1.07) 0.069	6.49 (5.32, 7.65) <0.001	8.93 (7.43, 10.44) <0.001	1.25 (0.56, 1.95) <0.001
Obesity	3.70 (3.03, 4.37) <0.001	1.33 (0.58, 2.07) 0.001	20.27 (18.70, 21.84) <0.001	20.05 (18.02, 22.09) <0.001	3.51 (2.58, 4.44) <0.001
High waist circumference	2.15 (1.61, 2.69) <0.001	1.29 (0.69, 1.90) <0.001	5.36 (4.08, 6.64) <0.001	3.16 (1.52, 4.81) <0.001	2.41 (1.65, 3.17) <0.001
Metabolic factors					
Elevated SBP	1.17 (0.59, 1.74) <0.001	6.71 (6.07, 7.35) <0.001	4.41 (3.06, 5.76) <0.001	30.34 (28.59, 32.09) <0.001	9.42 (8.61, 10.22) <0.001
Elevated fasting glucose	3.78 (2.75, 4.82) <0.001	4.63 (3.47, 5.78) <0.001	52.24 (49.62, 54.86) <0.001	12.75 (9.60, 15.91) <0.001	3.93 (2.46, 5.39) <0.001
Elevated TG	1.75 (0.96, 2.54) <0.001	2.42 (1.54, 3.29) <0.001	10.09 (8.22, 11.95) <0.001	6.96 (4.54, 9.38) <0.001	2.39 (1.29, 3.49) <0.001
Low HDL	1.10 (0.37, 1.83) 0.003	0.60 (−0.22, 1.41) 0.151	6.96 (5.22, 8.70) <0.001	1.96 (−0.27, 4.19) 0.085	1.77 (0.75, 2.79) 0.001
Elevated LDL	2.02 (1.26, 2.78) <0.001	−0.68 (−1.53, 0.17) 0.118	1.40 (−0.39, 3.19) 0.124	2.71 (0.40, 5.01) 0.022	2.44 (1.38, 3.51) <0.001
Lifestyle factor					
Current smoker	1.39 (0.20, 2.58) 0.023	4.03 (2.70, 5.36) <0.001	1.27 (−1.56, 4.10) 0.379	5.57 (1.85, 9.28) 0.003	3.59 (1.93, 5.26) <0.001

Data are presented as adjusted *β* coefficients with 95% confidence intervals estimated from multivariable generalized linear models. Reference categories were age 35–49 years, female sex, administrative and other staff, normal weight, normal waist circumference, normal systolic blood pressure, normal fasting glucose, normal triglycerides, normal HDL cholesterol, normal LDL cholesterol, and non-current smoker. Disease-specific analytical sample sizes were CAD, *n* = 1,915; stroke, *n* = 1,913; T2DM, *n* = 1,900; HTN, *n* = 1,855; and MACE, *n* = 1,900.

Significant interaction effects were observed for CAD, stroke, HTN, and MACE, whereas no significant interactions were identified for T2DM (Table 3). All reported interaction effects remained statistically significant after Benjamini–Hochberg false discovery rate (FDR) correction for multiple testing (adjusted *p* < 0.05). For CAD, age significantly modified the associations of BMI and occupation with predicted CAD risk, with the strongest interaction observed between age 60–70 years and obesity (*β* = 14.72, 95% CI: 12.99–16.44). Stroke risk was significantly influenced by interactions between age and elevated systolic blood pressure, age and male sex, and elevated systolic blood pressure and hyperglycemia, with the largest effect observed for age 60–70 years and male sex (*β* = 18.98, 95% CI: 17.40–20.56). For HTN, significant interactions were identified between age and BMI, sex and BMI, and BMI and nursing occupation. The effective subgroup sizes for the BMI × nursing occupation interactions were 154 overweight nurses and 188 nurses with obesity; these subgroup-based interaction estimates should nevertheless be interpreted cautiously. For MACE, significant interactions were observed between age and sex, age and BMI, and male sex and obesity, indicating that the effects of sex and adiposity on predicted cardiovascular risk varied across age groups.

**Table 3:** Significant interaction effects identified in multivariable generalized linear models for predicted cardiometabolic disease risks.

Disease	Significant interaction	*β* (95% CI)	*p* value
CAD	Age 60–70 × Obesity	14.72 (12.99, 16.44)	<0.001
	Age 60–70 × Overweight	5.19 (3.52, 6.87)	<0.001
	Age 50–59 × Obesity	3.90 (2.81, 4.98)	<0.001
	Age 50–59 × Overweight	2.17 (1.16, 3.17)	<0.001
	Age 60–70 × Physician	7.14 (5.15, 9.12)	<0.001
	Age 60–70 × Allied health professionals	3.22 (0.87, 5.57)	0.007
Stroke	Age 60–70 × High SBP	10.64 (9.07, 12.21)	<0.001
	Age 50–59 × High SBP	4.73 (3.52, 5.93)	<0.001
	Age 60–70 × Male	18.98 (17.40, 20.56)	<0.001
	Age 50–59 × Male	5.78 (4.80, 6.77)	<0.001
	High SBP × Hyperglycemia	4.52 (2.36, 6.67)	<0.001
T2DM	No significant interaction	—	—
HTN	Age 50–59 × Overweight	3.29 (0.06, 6.52)	0.046
	Age 60–70 × Obesity	−7.37 (−13.13, −1.60)	0.012
	Male × Overweight	−5.44 (−9.35, −1.52)	0.007
	Male × Obesity	−18.39 (−22.58, −14.19)	<0.001
	Overweight × Nursing staff	−3.98 (−7.92, −0.05)	0.047
	Obesity × Nursing staff	−3.99 (−7.89, −0.08)	0.045
MACE	Age 50–59 × Male	6.41 (5.05, 7.76)	<0.001
	Age 60–70 × Male	15.62 (13.58, 17.67)	<0.001
	Age 50–59 × Overweight	2.48 (1.06, 3.90)	0.001
	Age 50–59 × Obesity	1.70 (0.16, 3.24)	0.031
	Age 60–70 × Overweight	4.46 (2.06, 6.86)	<0.001
	Age 60–70 × Obesity	12.01 (9.66, 14.36)	<0.001
	Male × Obesity	−2.51 (−3.88, −1.14)	<0.001

Only statistically significant interaction terms (*p* < 0.05) are presented. No significant interaction effects were identified for T2DM (all *p* > 0.05). *β* values represent adjusted regression coefficients estimated from multivariable generalized linear models. Disease-specific analytical sample sizes were CAD, *n* = 1,915; stroke, *n* = 1,913; T2DM, *n* = 1,900; HTN, *n* = 1,855; and MACE, *n* = 1,900. Benjamini–Hochberg false discovery rate (FDR) correction was applied to account for multiple testing, and all reported interaction effects remained statistically significant (adjusted *p* < 0.05). For the HTN interaction analyses involving nursing occupation, the effective subgroup sizes were 154 overweight nurses and 188 nurses with obesity; these subgroup-based interaction estimates should be interpreted cautiously.

### 3.4 Association between occupational categories and cardiometabolic outcomes

In the age- and sex-adjusted ordinal logistic regression (OLR) analysis, both sex and age were significantly associated with predicted risk categories across all five cardiometabolic outcomes (Supplementary Table S3). Male employees exhibited significantly higher risks than female employees across all five outcomes (all *p* < 0.001). For HTN, male employees had higher odds of being classified into a higher predicted risk category than female employees (OR = 4.18, 95% CI: 3.21–5.44, *p* < 0.001). Increasing age was likewise associated with higher predicted risk categories across all five outcomes.

Regarding occupational categories, the age- and sex-adjusted associations varied across disease outcomes. Nurses had a significantly higher odds of being in a higher predicted risk category for T2DM (*p* = 0.036) and HTN (*p* = 0.024) than the administrative staff; thus, nursing work characteristics may have a potential link to metabolic risk (Supplementary Table S3). Conversely, physicians showed significantly lower risks for stroke (*p* = 0.031), HTN (*p* = 0.001), and MACE (*p* < 0.001). Although allied health professionals also exhibited lower odds of being in a higher predicted MACE risk category, the association did not reach statistical significance (*p* = 0.057). For CAD, no significant occupational differences were noted. Overall, the age- and sex-adjusted associations between occupational category and predicted cardiometabolic risk varied across disease outcomes.

### 3.5 Association between occupational categories and predicted risk of chronic diseases

Differences in the predicted 10-year risk of five major chronic diseases between occupational categories were evaluated using GLM, with adjustment for age and sex. Figure 3 illustrates the age- and sex-adjusted estimated marginal means, while the corresponding group-specific estimates with 95% CIs and pairwise comparisons are provided in Supplementary Tables S4, S5. Overall, no significant differences were observed for CAD, with adjusted predicted risks ranging from 5.84% (95% CI, 5.35–6.33) among allied health professionals to 6.57% (95% CI, 6.21–6.93) among nurses (Figure 3A; Supplementary Table S4).

**Figure 3:**
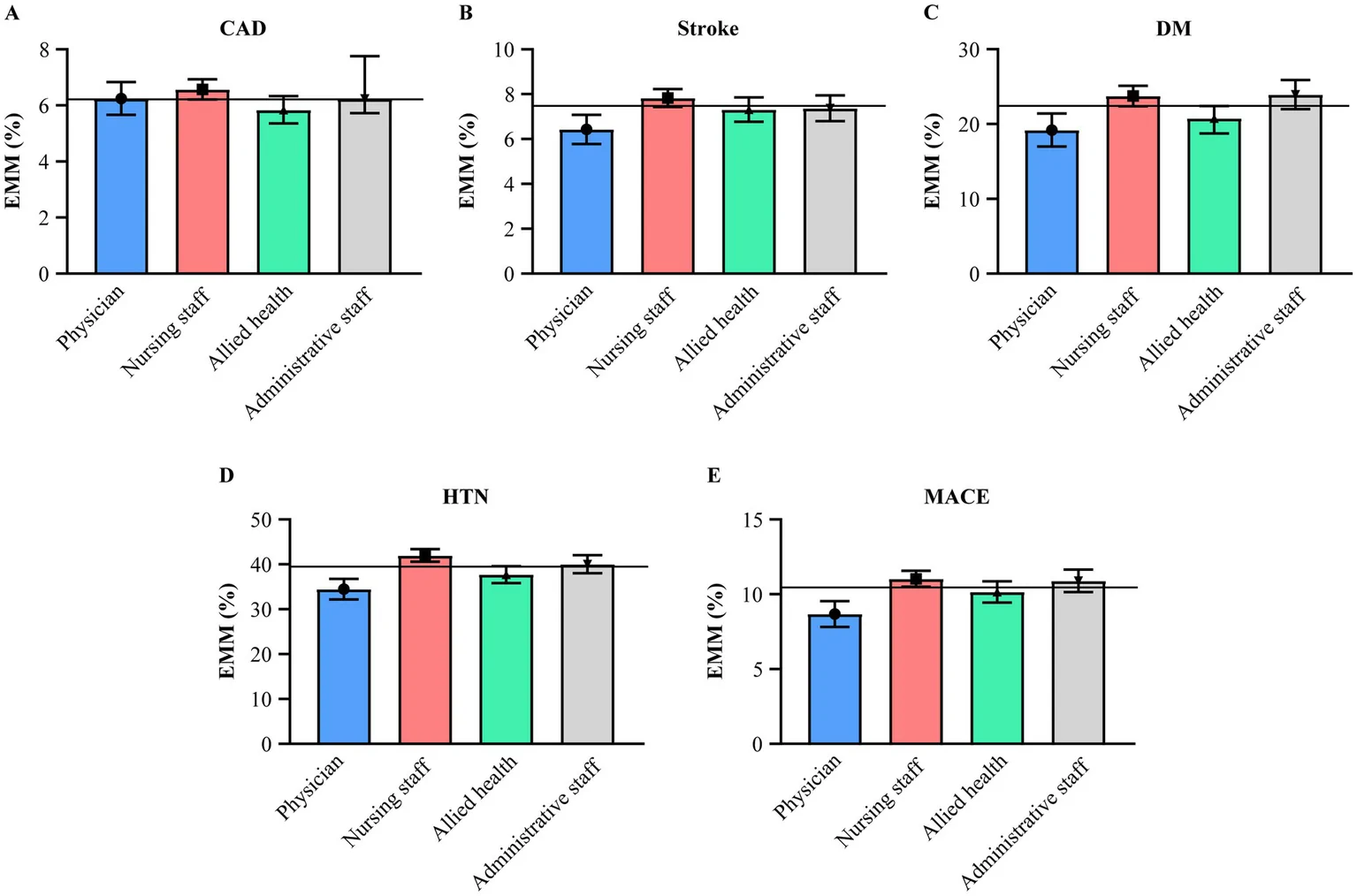
Occupational groups included physicians, nursing staff, allied health professionals, and administrative staff. **(A)** Coronary artery disease (CAD); **(B)** stroke; **(C)** type 2 diabetes mellitus (T2DM); **(D)** hypertension (HTN); **(E)** major adverse cardiovascular events (MACE).

Conversely, significant age- and sex-adjusted occupational disparities were observed for several outcomes (Figures 3B–E). Predicted stroke risk was higher among nursing staff [7.83% (95% CI, 7.43–8.23)] than among physicians [6.43% (95% CI, 5.78–7.08)], with a mean difference of 1.40% (95%CI, 0.27–2.52; adjusted *p* = 0.006) (Figure 3B; Supplementary Tables S4, S5). For T2DM, physicians had a lower adjusted predicted risk [19.20% (95% CI, 16.98–21.42)] than nurses [23.75% (95% CI, 22.39–25.11)] and administrative and other staff [23.94% (95% CI, 21.98–25.90)]. The corresponding mean differences were 4.55 percentage points (95% CI, 0.72–9.38; adjusted *p* = 0.010) and 4.74 percentage points (95% CI, 0.68–8.80; adjusted *p* = 0.013), respectively. Nurses also had a higher adjusted predicted risk than allied health professionals [20.57% (95% CI, 18.73–22.42)], with a mean difference of 3.18 percentage points (95% CI, 0.04–6.32; adjusted *p* = 0.046) (Figure 3C; Supplementary Tables S4, S5).

For HTN exhibited a similar pattern, where physicians had the lowest adjusted predicted risk (34.46%) and nursing (42.00%) and administrative staff (40.04%) demonstrated significantly higher adjusted predicted risks. The corresponding differences were 7.53 percentage points (95% CI, 3.58–11.49; adjusted *p* < 0.001) and 5.58 percentage points (95% CI, 1.38–9.77; adjusted *p* = 0.003), respectively. (Figure 3D; Supplementary Tables S4, S5). For MACE, physicians had the lowest adjusted predicted risk [8.68% (95% CI, 7.82–9.54)], compared with nurses [11.04% (95% CI, 10.51–11.57)], allied health professionals [10.16% (95% CI, 9.44–10.87)], and administrative and other staff [10.90% (95% CI, 10.14–11.66)]. Pairwise comparisons showed lower predicted risk among physicians than among nurses [mean difference, 2.35 percentage points (95% CI, 0.87–3.84); adjusted *p* < 0.001] and administrative and other staff [2.22 percentage points (95% CI, 0.64–3.79); adjusted *p* = 0.001] (Figure 3E; Supplementary Tables S4, S5).

Overall, physicians tended to be lower age- and sex-adjusted estimated marginal mean risks, particularly for T2DM, HTN, and MACE, whereas nursing and administrative staff tended to have higher adjusted predicted risks for these outcomes. However, the age- and sex-adjusted associations between occupational category and predicted cardiometabolic risk varied across disease outcomes, with no significant occupational differences observed for CAD.

### 3.6 Decision tree analysis across five Cardiometabolic outcomes

For CART analyses, intermediate- and high-risk participants were combined into a single higher-risk category because of the limited sample size in these groups. Our decision tree analysis revealed distinct yet partially overlapping risk stratification patterns across the five cardiometabolic diseases in both the overall population (Figure 4) and nursing staff (Figure 5). In both analyses, age consistently emerged as the primary splitting variable for CAD. Individuals older than 51–52.5 years exhibited a markedly higher likehood of being predicted into the higher-risk category than younger ones (Figures 4A, 5A). Further stratification by waist circumference demonstrated that central obesity substantially increased CAD risk. Notably, individuals with the highest risk, defined by advanced age and elevated waist circumference, 95.9% of the overall population and 87.5% of nurses were classified into the higher-risk group for CAD. These terminal-node percentages represent the proportion of participants classified into the higher-risk group within each node rather than individual predicted probabilities of CAD. The CART models were internally validated using 10-fold cross-validation. In the overall population, classification accuracy ranged from 86.9 to 91.7% across the five outcomes, while F1-scores ranged from 0.622 to 0.936. Sensitivity ranged from 47.7 to 92.3% and specificity from 80.2 to 99.5%, indicating variation in class-specific performance across outcomes. In particular, the CAD and stroke models showed relatively low sensitivity (55.3 and 47.7%, respectively) despite high specificity (99.5 and 98.7%, respectively). Detailed performance metrics are provided in Supplementary Table S6.

**Figure 4:**
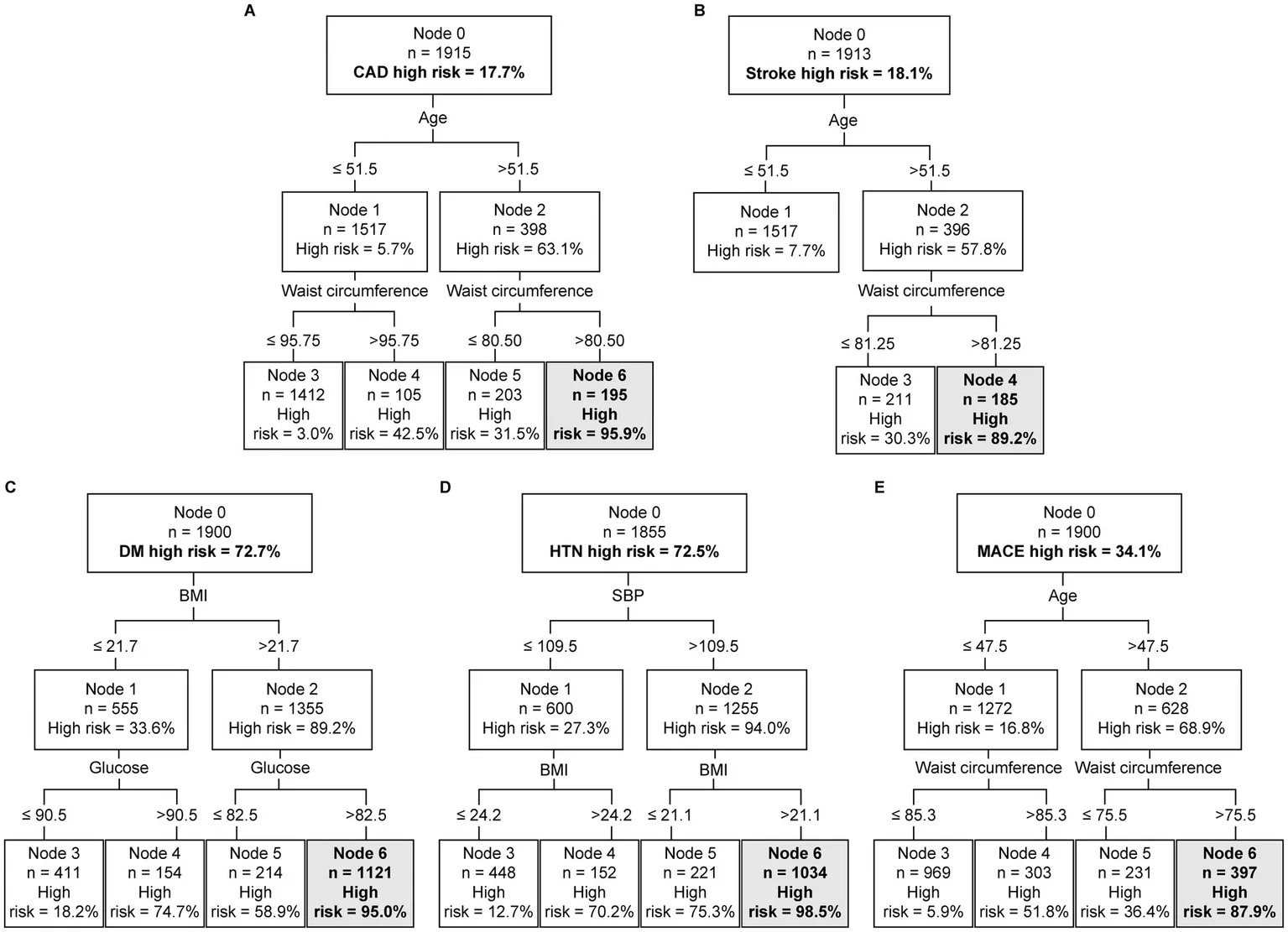
Decision tree–based risk stratification of five cardiometabolic diseases. Decision tree models identified key determinants of high-risk status for **(A)** CAD, **(B)** stroke, **(C)** T2DM, **(D)** HTN, and **(E)** MACE. Each node displays the sample size and proportion of high-risk individuals. Age was the primary splitting variable in CAD, stroke, and MACE, whereas BMI and SBP were dominant in T2DM and HTN, respectively. Subsequent splits were mainly driven by adiposity-related and metabolic factors. Terminal nodes represent distinct subgroups with a progressive increase in the proportion of participants classified into the higher-risk category. CAD, coronary artery disease; T2DM, Type 2 diabetes mellitus; HTN, hypertension; MACE, major adverse cardiovascular events.

**Figure 5:**
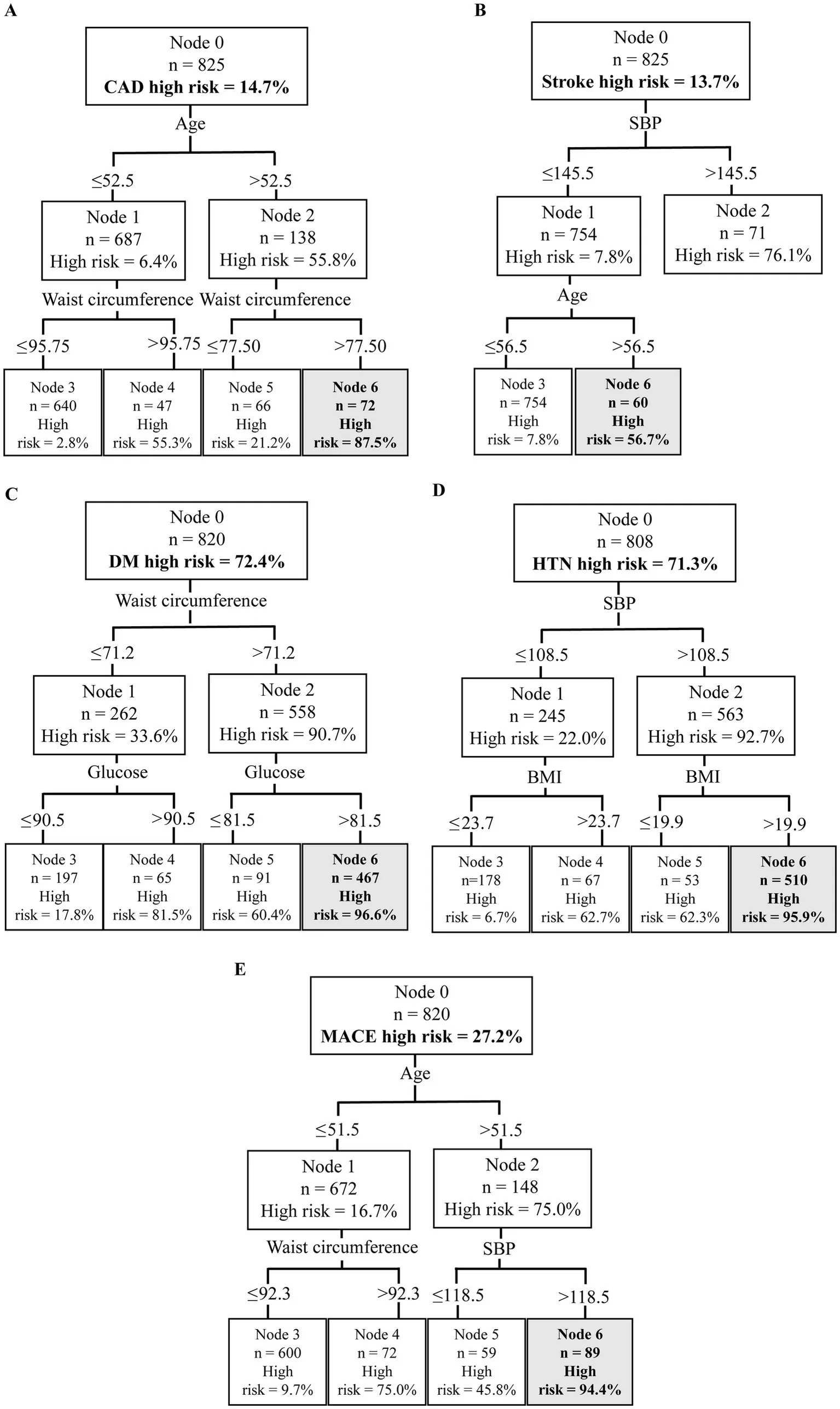
Decision tree–based risk stratification of five cardiometabolic diseases in nurses. Decision tree models identified key determinants of high-risk status for **(A)** coronary artery disease (CAD), **(B)** stroke, **(C)** Type 2 Diabetes Mellitus (T2DM), **(D)** Hypertension (HTN), and **(E)** Major Adverse Cardiovascular Events (MACE). Each node displays the sample size and proportion of high-risk individuals. Terminal nodes represent distinct subgroups with a progressively increasing proportion of higher-risk cases.

Similarly, for stroke, age served as the initial determinant in the overall population (Figure 4B), whereas SBP emerged as the primary splitting variable in nurses (Figure 5B). However, in both analyses, additional factors, including waist circumference and age, further refined stroke risk, indicating an interaction between hemodynamic and demographic characteristics. Individuals with elevated SBP or advanced age consistently demonstrated substantially higher proportions of higher-risk cases, with terminal nodes reaching 76–89% classification into the higher-risk group.

Conversely, for T2DM, metabolic factors played a dominant role. BMI was the primary splitting variable in the overall population (Figure 4C), whereas waist circumference was the initial determinant in nurses (Figure 5C). Despite this difference, both models consistently identified adiposity and glucose levels as key contributors to risk stratification. Individuals with both elevated adiposity measures and fasting glucose exhibited markedly increased disease prevalence in top node, with the highest-risk subgroups reaching 95–97% classified into the higher-risk group.

For HTN, SBP was the primary determinant in both overall populations and nurses (Figures 4D, 5D). Individuals with elevated SBP showed substantially higher likehood of being classified into the higher-risk group, which was further modified by BMI. Those with both higher SBP and BMI were consistently identified as the highest-risk subgroup, with over 95% of individuals in these terminal nodes classified into the higher-risk category. Finally, for MACE, age was the primary splitting variable in both populations (Figures 4E, 5E). Older individuals exhibited a higher rate of higher-risk classification, which was further stratified by waist circumference and/or SBP. Individuals classified into the highest-risk subgroup for MACE were those with both advanced age and adverse metabolic profiles.

Overall, these findings demonstrate a high degree of consistency between the overall population and the nursing staff, particularly in the identification of age and adiposity-related measures as key determinants of risk. However, some differences, such as the greater role of SBP in stroke among nurses and the use of waist circumference instead of BMI in T2DM, were noted, suggesting that risk factors vary by occupation.

## 4 Discussion

This study characterized the predicted 10-year cardiometabolic risk scores for five chronic diseases (CAD, stroke, T2DM, HTN, and MACE) among hospital staff and examined their associations with demographic, behavioral, clinical, and occupational factors. Age and sex were strongly associated with the predicted 10-year risk scores for five chronic diseases (CAD, stroke, T2DM, HTN, and MACE), with male participants showing higher stroke and MACE risks. Multivariable analyses further demonstrated that increasing age was consistently associated higher predicted risk scores across all five outcomes, whereas male sex was associated with higher predicted risk scores of CAD, stroke, and MACE but lower predicted risk score of T2DM and HTN after adjustment for other metabolic factors. These patterns are generally consistent with prior epidemiological evidence indicating a higher incidence of cardiovascular events in males (34, 35). Importantly, our findings further highlight the heterogeneous nature of cardiometabolic risk profiles. Adverse outcomes may synergistically increase in individuals with multiple risk factors, reflecting the nonlinear interactions among risk factors and underscoring the importance of integrated risk models rather than single-factor evaluation (36).

Notably, our findings further suggest that the impact of metabolic abnormalities may differ by sex. Females with T2DM or HTN may experience a disproportionately greater increase in cardiovascular risk, particularly in those at the postmenopausal stage (37, 38). These findings agree with previous studies reporting sex-specific differences in cardiovascular risk profiles and outcomes. A recent analysis from the UK Biobank, including over 470,000 participants, reported that although the overall stroke incidence was higher in males, several risk factors exhibited stronger associations with stroke outcomes in females (37). Likewise, a previous study have demonstrated that atherogenic lipid profiles are associated with blood pressure dysregulation, with females showing disproportionately stronger effects, thereby highlighting sex-specific metabolic susceptibility (39). These observations may be partly explained by biological mechanisms, including estrogen’s protective effects in premenopausal females, as well as differences in risk factor exposure and healthcare utilization (40). Additionally, prior large-scale cohort studies have demonstrated that certain risk factors may exert stronger effects in females, underscoring the complexity of sex-specific risk patterns (38, 41, 42). However, the inverse adjusted associations of male sex with the continuous predicted T2DM and HTN risk scores should be interpreted cautiously. Male participants had less favorable measured metabolic profiles and showed higher odds of being classified into higher T2DM and HTN predicted risk categories in the ordinal logistic regression analyses. Therefore, the inverse associations observed in the generalized linear models should not be interpreted as indicating an overall lower T2DM or HTN risk in males. Rather, they represent conditional associations with the continuous platform-derived risk scores after adjustment for the included metabolic factors and may reflect differences in outcome scale and model specification.

An important methodological consideration is that the predicted 10-year risk scores were generated by a risk assessment platform that incorporates several of the same clinical indicators examined as predictors in our analyses, including blood pressure, fasting glucose, blood lipids, and BMI (33). Consequently, associations between these variables and the predicted risk scores are partly inherent to the construction of the risk algorithm and should not be interpreted as independent or novel determinants of disease risk. In particular, the relatively large effect estimates observed for some clinical indicators may reflect the sensitivity of the platform-derived risk scores to their input components rather than independent epidemiological associations. Therefore, these analyses should primarily be interpreted as characterizing patterns within the platform-derived risk estimates.

Moreover, this study found that BMI, blood pressure, and metabolic biomarkers were strongly associated with all of the five major disease outcomes, reinforcing the central role of metabolic dysfunction in chronic disease development (43). In line with our findings, global burden analyses showed that elevated BMI significantly contributes to ischemic stroke mortality and disability, particularly in regions with rising cardiometabolic risk (44). Notably, male participants exhibited more adverse metabolic profiles, including higher BMI, blood pressure, glucose, triglyceride, and LDL-C levels, along with lower HDL-C levels, than female participants. Our decision tree analysis further revealed that SBP and fasting plasma glucose are key discriminators of high-risk groups. In addition, age, central obesity, and metabolic indicators repeatedly emerged as primary splitting nodes across disease models, suggesting nonlinear interactions and threshold effects. Therefore, early identification of metabolic abnormalities before the onset of clinically overt disease is important. Increasing age remained a major predictor across all disease outcomes, consistent with existing literature showing that aging is an independent risk factor for CVD and is often compounded by comorbid conditions such as obesity and T2DM (45). Furthermore, while chronological age has been increasingly reported as a universal risk predictor across populations, advanced imaging-based models suggest that age-related structural and functional alterations in the cardiovascular system underlie the increased risk of developing CAD, heart failure, and stroke (46). Age-related biological changes, together with prolonged exposure to environmental, behavioral, and metabolic risk factors, may contribute to the increased cardiometabolic risk observed with agin (47). Importantly, our findings from both the multivariable and interaction analyses suggest the association between age and predicted cardiometabolic risk may vary according to metabolic factors, particularly adiposity. Significant age-by-BMI interactions observed for CAD and HTN further indicate that the association between age and predicted risk may differ across BMI levels.

The association between occupational category and cardiometabolic risk varied according to the analytical approach and disease outcome, indicating that occupation alone may not be a consistent independent predictor but may be associated with specific outcomes in conjunction with metabolic and work-related factors. These apparently differing findings across models likely reflect differences in the outcome scale and analytical approach. The generalized linear models evaluated continuous predicted risk scores, whereas the ordinal logistic regression models evaluated the likelihood of being classified into higher predicted risk categories. Thus, an occupational group may differ in the probability of crossing clinically defined risk-category thresholds without showing a substantial difference in the mean continuous predicted risk score after adjustment. Although significant differences in disease risk were observed across occupational groups in the unadjusted analyses, occupational category was not independently associated with predicted cardiometabolic risk after adjustment for demographic and metabolic factors in the multivariable generalized linear models. Nevertheless, the ordinal logistic regression analysis showed that nursing occupation was associated with higher predicted risks of T2DM and HTN, whereas physician occupation was associated with lower predicted risks of stroke, HTN, and MACE, suggesting that associations between occupational category and predicted disease risk may vary across disease outcomes. Accordingly, the findings from the two models should be considered complementary rather than directly contradictory, as they characterize different aspects of the platform-derived predicted risk estimates.

Our findings are broadly consistent with previous studies of hospital employees in Taiwan. Yeh et al. reported occupational differences in MetS prevalence, with higher prevalence among physicians and administrative staff, while Ho et al. identified male sex, older age, and administrative occupation as factors associated with MetS (26, 27). Tsai and Tsou also reported age-, sex-, and occupation-related differences in MetS among Taiwanese healthcare workers (28). In addition, an 8-year cohort study of Taiwanese hospital workers by Cheng et al. found that night-shift work was associated with incident MetS and elevated waist circumference (29). Collectively, these studies indicate that demographic, metabolic, and occupational characteristics are relevant to cardiometabolic health among hospital employees, broadly supporting the patterns observed in our study. Nevertheless, direct comparisons should be interpreted cautiously because previous studies primarily evaluated MetS and its components, whereas our study examined platform-derived 10-year predicted risks across five cardiometabolic outcomes. Previous studies have reported associations between occupational characteristics and cardiovascular and metabolic risk (16, 17, 48, 49). However, specific occupational exposures, including shift work, working hours, and psychosocial stress, were not measured in the present study; therefore, their potential contributions to the observed occupational differences cannot be determined. Furthermore, the significant interaction between BMI and nursing occupation observed in our interaction analysis suggests that the association between BMI and predicted risk may vary by occupational category; however, the underlying mechanisms cannot be determined from the present data. Therefore, the observed occupational differences should be interpreted as associations with platform-derived predicted risk estimates rather than effects of specific workplace exposures.

This study has several strengths. First, the cohort of 1,916 hospital employees allowed the simultaneous characterization of platform-derived 10-year predicted risks across five cardiometabolic outcomes. Second, the inclusion of demographic, behavioral, clinical, and occupational factors enabled the assessment of cardiometabolic risk patterns from multiple perspectives. Third, the use of complementary analytical approaches, including generalized linear models, ordinal logistic regression, interaction analyses, and CART, allowed different aspects of the predicted risk profiles to be examined.

This study also has several limitations. First, the cross-sectional design precludes causal inference, and the single-center setting may limit the generalizability of the findings. Second, the outcomes were 10-year predicted risks generated by the National Health Administration Chronic Disease Risk Assessment Platform rather than observed disease events. Because several clinical predictors (e.g., blood pressure, fasting glucose, blood lipids, and BMI) are also incorporated into the prediction algorithm, their regression coefficients may partly reflect the internal structure of the risk model and should not be interpreted as independent etiological effects. Moreover, the predicted risks were not validated against longitudinal incident disease outcomes or in an independent external cohort, and residual confounding from unmeasured factors cannot be excluded. Information on medication use was unavailable; therefore, the potential effects of antihypertensive, glucose-lowering, or lipid-lowering medications on clinical and biochemical measurements could not be evaluated. Finally, some subgroup analyses involved small sample sizes, particularly among male nurses (*n* = 16) and underweight males (*n* = 4), and the BMI-by-nursing occupation interaction analyses should therefore be interpreted cautiously. Future prospective, multicenter studies with larger and more diverse populations are needed to validate these findings against observed incident disease outcomes.

In conclusion, this study characterized platform-estimated 10-year cardiometabolic disease risk patterns among employees of a single medical center. Differences in predicted risk estimates were observed across demographic, metabolic, and occupational characteristics, with some interaction patterns identified among these factors. These findings represent associations with platform-derived risk estimates and should not be interpreted as evidence that these factors causally influence future disease occurrence. The integration of traditional regression models with decision tree–based approaches provided complementary information for characterizing predicted risk patterns and identifying employee subgroups with higher estimated risk profiles. These findings suggest that platform-derived risk estimates may provide supplementary information for cardiometabolic risk assessment and workplace health management among hospital employees. However, whether integration of such risk assessment tools into routine employee health examinations improves risk stratification, preventive management, or clinical outcomes remains to be determined and warrants further prospective evaluation.

## Data Availability

The datasets presented in this article are not readily available because the datasets are not publicly available due to institutional and ethical restrictions. Requests to access the datasets should be directed to the corresponding author and are subject to approval by the Institutional Review Board and institutional policies. Requests to access the datasets should be directed to C-FC, cfchang.gina@gmail.com.
