# Supplementary material for: Predicted 10-year cardiometabolic risk among hospital staff: a cross-sectional study
Source: Front Public Health. 2026 Sep 16;14:1929290. doi: 10.3389/fpubh.2026.1929290 (PMC13624112; doi:10.3389/fpubh.2026.1929290)
Supplement: Supplementary file 1 [file Data_Sheet_1.docx]

Supplementary Material

# 1 Supplementary Tables

**Supplementary Table 1.** Comparison of data completeness according to sex and occupational category.

| Panel A. By sex | Incomplete, n (%) | Complete, n (%) | Total | P-value |
| --- | --- | --- | --- | --- |
| Male | 24 (2.70) | 864 (97.30) | 888 | 0.764 |
| Female | 64 (2.52) | 2,478 (97.48) | 2,542 |  |
| Total | 88 | 3,342 | 3,430 |  |

Pearson’s χ² = 0.090.

| Panel B. By occupational category | Incomplete, n (%) | Complete, n (%) | Total | P-value |
| --- | --- | --- | --- | --- |
| Physicians | 19 (2.77) | 668 (97.23) | 687 | 0.028 |
| Nursing staff | 50 (3.27) | 1,481 (96.73) | 1,531 |  |
| Allied health | 13 (2.03) | 628 (97.97) | 641 |  |
| Administrative staff | 6 (1.05) | 565 (98.95) | 571 |  |
| Total | 88 | 3,342 | 3,430 |  |

Pearson’s χ² = 9.095, df = 3.

*Post-hoc pairwise comparisons with Benjamini–Hochberg FDR correction showed a significantly higher proportion of incomplete records among nursing staff than administrative/other staff (3.27% vs. 1.05%; FDR-adjusted P = 0.030). No other pairwise comparisons remained statistically significant after correction.

**Supplementary Table 2.** Univariable generalized linear models for predicted cardiometabolic disease risks.

| **Variable** | **CAD**  **β (95% CI)** | **Stroke**  **β (95% CI)** | **T2DM**  **β (95% CI)** | **HTN**  **β (95% CI)** | **MACE**  **β (95% CI)** |
| --- | --- | --- | --- | --- | --- |
| **Demographic factors** |  |  |  |  |  |
| **Age** |  |  |  |  |  |
| Age 50–59 | 6.14  (5.57, 6.70) | 6.03  (5.39, 6.67) | 8.76  (6.71, 10.80) | 19.75  (17.44, 22.06) | 8.67  (7.76, 9.58) |
| Age 60–70 | 18.99  (18.05, 19.94) | 19.30  (18.22, 20.38) | 19.46  (15.94, 22.98) | 40.60  (36.55, 44.64) | 25.62  (24.09, 27.16) |
| **Sex** |  |  |  |  |  |
| Male | 3.60  (2.88, 4.31) | 5.33  (4.58, 6.08) | 4.86  (2.89, 6.83) | -0.19  (-2.66, 2.28) | 9.10  (8.09, 10.11) |
| **Occupation** |  |  |  |  |  |
| Physician | 1.61  (0.58, 2.64) | 1.83  (0.72, 2.93) | -1.21  (-4.02, 1.61) | -5.58  (-9.05, -2.11) | 2.68  (1.16, 4.21) |
| Nurse | -1.03  (-1.92, -0.15) | -1.31  (-2.25, -0.36) | -2.34  (-4.75, 0.07) | -1.07  (-4.01, 1.87) | -2.77  (-4.07, -1.47) |
| Allied health  professionals | -1.69  (-2.70, -0.67) | -1.03  (-2.12, 0.07) | -4.18  (-6.96, -1.40) | -6.77  (-10.17, -3.37) | -1.81  (-3.31, -0.31) |
| **Anthropometric measures** |  |  |  |  |  |
| Underweight | -1.96  (-3.60, -0.32) | -2.00  (-3.84, -0.15) | -4.54  (-8.27, -0.81) | -8.38  (-13.34, -3.43) | -3.10  (-5.55, -0.64) |
| Overweight | 3.51  (2.80, 4.23) | 2.99  (2.18, 3.79) | 10.33  (8.69, 11.96) | 12.56  (10.35, 14.77) | 5.07  (3.99, 6.15) |
| Obesity | 7.19  (6.46, 7.93) | 5.57  (4.75, 6.39) | 30.62  (28.94, 32.30) | 31.38  (29.12, 33.64) | 10.11  (9.01, 11.21) |
| High waist  circumference | 5.66  (5.03, 6.28) | 4.23  (3.53, 4.93) | 21.80  (20.26, 23.34) | 23.23  (21.25, 25.22) | 7.34  (6.39, 8.28) |
| **Metabolic factors** |  |  |  |  |  |
| Elevated SBP | 6.10  (5.19, 7.01) | 10.70  (9.80, 11.60) | 16.75  (14.29, 19.21) | 43.19  (40.67, 45.71) | 15.45  (14.22, 16.68) |
| Elevated fasting  glucose | 9.47  (7.75, 11.19) | 10.15  (8.30, 11.99) | 64.34  (60.04, 68.64) | 29.32  (23.52, 35.11) | 12.19  (9.58, 14.80) |
| Elevated TG | 6.65  (5.41, 7.89) | 7.16  (5.83, 8.49) | 27.39  (24.11, 30.66) | 24.02  (19.85, 28.19) | 10.54  (8.69, 12.38) |
| Low HDL | 5.02  (3.88, 6.17) | 4.77  (3.54, 6.00) | 24.31  (21.33, 27.29) | 14.30  (10.45, 18.15) | 8.26  (6.56, 9.95) |
| Elevated LDL | 3.28  (1.96, 4.6) | 0.61  (-0.81, 2.04) | 6.74  (3.16, 10.32) | 6.93  (2.50, 11.35) | 4.56  (2.60, 6.53) |
| **Lifestyle factor** |  |  |  |  |  |
| Current smoker | 6.87  (4.89, 8.85) | 10.02  (7.92, 12.12) | 10.61  (5.14, 16.09) | 13.78  (6.89, 20.67) | 13.43  (10.52, 16.33) |

Notes: β values represent regression coefficients from univariable generalized linear models. Disease-specific analytical sample sizes were CAD, n = 1,915; stroke, n = 1,913; T2DM, n = 1,900; HTN, n = 1,855; and MACE, n = 1,900. Positive β values indicate higher predicted disease risk relative to the reference group, whereas negative β values indicate lower predicted disease risk. Reference groups were age 35–49 years, female sex, administrative and other staff, normal weight (BMI 18.5–23.9 kg/m²), normal waist circumference, normal systolic blood pressure, normal fasting glucose, normal triglycerides, normal HDL cholesterol, normal LDL cholesterol, and non-current smokers. CAD, coronary artery disease; T2DM, type 2 diabetes mellitus; HTN, hypertension; MACE, major adverse cardiovascular events; CI, confidence interval. circumference, normal systolic blood pressure, normal fasting glucose, normal triglycerides, normal HDL cholesterol, normal LDL cholesterol, and non-smokers.

**Supplementary Table 3.** Multivariable ordinal logistic regression analyses of age, sex, and occupational category associated with predicted cardiometabolic disease risk categories.

| **Disease** | **Variable** | **Adjusted OR (95% CI)** | **p-value** |
| --- | --- | --- | --- |
| CAD | Male (vs Female)  Age (per year)  Physician (vs Admin)  Nurse (vs Admin)  Allied health (vs Admin) | 2.45 (1.60, 3.74)  1.31 (1.28, 1.34)  1.00 (0.60, 1.65)  1.03 (0.68, 1.57)  0.71 (0.42, 1.17) | **< 0.001**  **< 0.001**  0.992  0.880  0.179 |
| Stroke | Male (vs Female)  Age (per year)  Physician (vs Admin)  Nurse (vs Admin)  Allied health (vs Admin) | 12.71 (8.18, 19.73)  1.29 (1.26, 1.32)  0.58 (0.35, 0.95)  1.45 (0.93, 2.25)  0.83 (0.50, 1.36) | **< 0.001**  **< 0.001**  **0.031**  0.099  0.455 |
| T2DM | Male (vs Female)  Age (per year)  Physician (vs Admin)  Nurse (vs Admin)  Allied health (vs Admin) | 2.43 (1.87, 3.16)  1.12 (1.11, 1.14)  0.74 (0.54, 1.03)  1.30 (1.02, 1.67)  0.89 (0.66, 1.18) | **< 0.001**  **< 0.001**  0.074  **0.036**  0.408 |
| HTN | Male (vs Female)  Age (per year)  Physician (vs Admin)  Nurse (vs Admin)  Allied health (vs Admin) | 4.18 (3.21, 5.44)  1.01 (1.00, 1.03)  0.59 (0.43, 0.81)  1.32 (1.04, 1.68)  0.84 (0.63, 1.11) | **< 0.001**  **0.015**  **0.001**  **0.024**  0.214 |
| MACE | Male (vs Female)  Age (per year)  Physician (vs Admin)  Nurse (vs Admin)  Allied health (vs Admin) | 14.34 (10.09, 20.37)  1.26 (1.23, 1.28)  0.46 (0.30, 0.69)  1.07 (0.77, 1.48)  0.69 (0.47, 1.01) | **< 0.001**  **< 0.001**  **< 0.001**  0.705  0.057 |

* Abbreviations: OR, odds ratio; CI, confidence interval. Admin, administrative staff. All models simultaneously included age, sex, and occupational category. Admin was used as the reference group for occupational categories. Female was used as the reference group for sex. Disease-specific analytical sample sizes were CAD, n = 1,915; stroke, n = 1,913; T2DM, n = 1,900; HTN, n = 1,855; and MACE, n = 1,900. Bold values indicate statistical significance (P < 0.05).

**Supplementary Table 4.** Age- and sex-adjusted estimated marginal means of predicted 10-year cardiometabolic risk across occupational categories.

| Disease | Physician, %  (95% CI) | Nurse, %  (95% CI) | Allied health, %  (95% CI) | Admin, %  (95% CI) |
| --- | --- | --- | --- | --- |
| CAD | 6.25  (5.66, 6.83) | 6.57  (6.21, 6.93) | 5.84  (5.35, 6.33) | 6.24  (5.72–6.75) |
| Stroke | 6.43  (5.78, 7.08) | 7.83  (7.43, 8.23) | 7.31  (6.77, 7.86) | 7.37  (6.79, 7.94) |
| T2DM | 19.20  (16.98, 21.42) | 23.75  (22.39, 25.11) | 20.57  (18.73, 22.42) | 23.94  (21.98, 25.90) |
| HTN | 34.46  (32.17, 36.75) | 42.00  (40.59, 43.40) | 37.71  (35.80, 39.61) | 40.04  (38.02, 42.06) |
| MACE | 8.68  (7.82, 9.54) | 11.04  (10.51, 11.57) | 10.16  (9.44, 10.87) | 10.90  (10.14, 11.66) |

* Disease-specific analytical sample sizes were CAD, n = 1,915; stroke, n = 1,913; T2DM, n = 1,900; HTN, n = 1,855; and MACE, n = 1,900.

**Supplementary Table 5.** Significant pairwise comparisons of adjusted predicted cardiometabolic disease risks across occupational categories after adjustment for age and sex.

| **Disease** | **Occupation 1** | **Occupation 2** | **Mean Difference (%)** | **95% CI** | **Adjusted**  **p-value** |
| --- | --- | --- | --- | --- | --- |
| Stroke | Physician | Nurse | -1.40 | (-2.52, -0.27) | 0.006 |
| T2DM | Physician  Physician  Nurse | Nurse  Admin  Allied health | -4.55  -4.74  3.18 | (-9.38, -0.72)  (-8.80, -0.68)  (0.04, 6.32) | 0.010  0.013  0.046 |
| HTN | Physician  Physician  Nurse | Nurse  Admin  Allied health | -7.53  -5.576  4.285 | (-11.49, -3.58)  (-9.77, -1.38)  (1.04, 7.53) | <0.001  0.003  0.003 |
| MACE | Physician  Physician  Physician | Nurse  Allied health  Admin | -2.353  -1.471  -2.215 | (-3.84, -0.87)  (-2.94, 0.00)  (-3.79, -0.64) | 0.000  0.050  0.001 |

* Models included occupational category, age, and sex, with age and sex included as adjustment covariates. Pairwise comparisons across occupational categories were based on age- and sex-adjusted estimated marginal means. Disease-specific analytical sample sizes were CAD, n = 1,915; stroke, n = 1,913; T2DM, n = 1,900; HTN, n = 1,855; and MACE, n = 1,900.

**Supplementary Table 6**. Performance of the 10-fold cross-validated CRT decision tree models for predicted 10-year cardiometabolic risk.

| Outcome | Accuracy  (%) | Sensitivity  (%) | Specificity  (%) | Precision  (%) | F1-score |
| --- | --- | --- | --- | --- | --- |
| CAD | 91.7 | 55.3 | 99.5 | 95.9 | 0.702 |
| Stroke | 89.5 | 47.7 | 98.7 | 89.2 | 0.622 |
| T2DM | 88.5 | 91.6 | 80.2 | 92.5 | 0.921 |
| HTN | 90.8 | 92.3 | 87.1 | 94.9 | 0.936 |
| MACE | 86.9 | 73.4 | 93.9 | 86.1 | 0.793 |

* The CRT models were internally validated using 10-fold cross-validation. For calculation of class-sensitive performance measures, the combined intermediate/high-risk category was considered the positive class and the low-risk category the negative class. Sensitivity represents the proportion of intermediate/high-risk individuals correctly classified, whereas specificity represents the proportion of low-risk individuals correctly classified. Disease-specific analytical sample sizes were CAD, n = 1,915; stroke, n = 1,913; T2DM, n = 1,900; HTN, n = 1,855; and MACE, n = 1,900.
